# Genetic predispositions for anxiety disorders and major depressive disorder affect current dietary habits in older patients with lifestyle-related diseases

**DOI:** 10.1093/ijnp/pyag014

**Published:** 2026-03-21

**Authors:** Kazutaka Ohi, Daisuke Nishizawa, Daisuke Fujikane, Junko Hasegawa, Naomi Sato, Fumihiko Tanioka, Haruhiko Sugimura, Kazutaka Ikeda, Toshiki Shioiri

**Affiliations:** Department of Psychiatry, Gifu University Graduate School of Medicine, Gifu, Japan; Addictive Substance Project, Tokyo Metropolitan Institute of Medical Science, Tokyo, Japan; Addictive Substance Project, Tokyo Metropolitan Institute of Medical Science, Tokyo, Japan; Department of Neuropsychopharmacology, National Institute of Mental Health, National Center of Neurology and Psychiatry (NCNP), Tokyo, Japan; Department of Psychiatry, Gifu University Graduate School of Medicine, Gifu, Japan; Addictive Substance Project, Tokyo Metropolitan Institute of Medical Science, Tokyo, Japan; Department of Clinical Nursing, Hamamatsu University School of Medicine, Shizuoka, Japan; Department of Pathology, Iwata City Hospital, Shizuoka, Japan; Sasaki Institute Sasaki Foundation, Tokyo, Japan; Addictive Substance Project, Tokyo Metropolitan Institute of Medical Science, Tokyo, Japan; Department of Neuropsychopharmacology, National Institute of Mental Health, National Center of Neurology and Psychiatry (NCNP), Tokyo, Japan; Department of Psychiatry, Gifu University Graduate School of Medicine, Gifu, Japan

**Keywords:** dietary intake, polygenic risk score, genome-wide association study, anxiety disorders, major depressive disorder

## Abstract

**Background:**

Dietary intake is critical for the prevention and management of physical and mental disorders and may also be influenced by genetic predispositions for anxiety disorders and major depressive disorder (MDD). This study examined the effects of polygenic risk scores (PRSs) for anxiety disorders and MDD on current dietary intake in older patients with lifestyle-related diseases.

**Methods:**

Current dietary intake was assessed across 8 categories: miso soup, Japanese tea, green and yellow vegetables, light-colored vegetables, fruits, pickles, meats, and soybeans in 730 older outpatients diagnosed with or suspected of having lifestyle-related diseases, such as hypertension, diabetes mellitus, and cancer, at Iwata City Hospital, Shizuoka, Japan, during the recruitment period from 2003 to 2008. Polygenic risk scores for anxiety disorders and MDD were calculated using large-scale genome-wide association studies datasets, and their correlations with dietary intake patterns in these patients were investigated. Furthermore, genetic correlations between these disorders and specific dietary intake were explored in an independent general cohort from the UK Biobank.

**Results:**

Higher PRSs for anxiety disorders were significantly correlated with increased fruit intake (*R^2^* = 0.010, *P* = 2.62 × 10^-3^) in these patients. Conversely, higher PRSs for MDD were nominally correlated with decreased intake of fruits (*R^2^* = 0.0056, *P* = .021), Japanese tea (*R^2^* = 0.0069, *P* = .014), green vegetables (*R^2^* = 0.0076, *P* = 7.82 × 10^-3^), light-colored vegetables (*R^2^* = 0.0049, *P* = .029), and soybeans (*R^2^* = 0.0054, *P* = .025) and with increased intake of meats (*R^2^* = 0.0082, *P* = 8.02 × 10^-3^). In contrast, in the independent general cohort, both disorders were commonly genetically correlated with decreased fruit intake (anxiety disorders: *r_g_* = -0.10, *P* = 2.10 × 10^-3^; MDD: *r_g_* = -0.17, *P* = 2.01 × 10^-9^).

**Conclusions:**

Our findings suggest that genetic factors related to anxiety disorders may shape dietary habits, potentially influencing compensatory healthy dietary behaviors in response to anxiety and worry related to physical health issues.

**Significance statement**

Genetic predispositions for anxiety disorders may contribute to compensatory healthy dietary behaviors in response to physical health-related anxiety, whereas those for major depressive disorder (MDD) may be related to unhealthy eating patterns in patients with lifestyle-related diseases. Genetic factors for anxiety disorders and MDD distinctly influence dietary behaviors.

## Introduction

Anxiety disorders and major depressive disorder (MDD) are among the most common and debilitating mental disorders worldwide, affecting approximately 7.3% and 4.4% of the population, respectively.^1^ These disorders frequently co-occur; approximately 50% of individuals diagnosed with depression also meet the criteria for an anxiety disorder.^2^^,^^3^ They share common pathophysiological features, such as dysregulation of neural circuits and neurotransmitter systems—including the serotonergic, noradrenergic, and dopaminergic pathways^4^—and exhibit neuroimaging abnormalities in brain areas involved in mood regulation, such as the amygdala, prefrontal cortex, and hippocampus.^5^ Additionally, both disorders also share genetic predispositions involving multiple genetic variants.^6^^,^^7^ In contrast, specific differences also exist, including variations in neural activity in the amygdala and hippocampus^8^ and genetic factors related to childhood sleep disturbances, such as disorders of arousal or nightmares and disorders of initiating and maintaining sleep.^9^ These similarities and specificities between the 2 disorders may be influenced by shared and distinct genetic, environmental, and psychological factors.

Nutritional psychiatry has identified certain dietary patterns that may exacerbate or alleviate symptoms associated with psychiatric disorders.^10^ Notable research has demonstrated prominent relationships between dietary intake patterns and the risk of developing anxiety disorders and MDD.^11–14^ High dietary intake of refined sugars and saturated fats can increase vulnerability to these disorders by promoting inflammatory processes and oxidative stress, which adversely affect brain function.^11–13^ Conversely, high intakes of fruits, vegetables, whole grains, fish, and omega-3 fatty acids—such as the Mediterranean, Norwegian, or Japanese diets—have been associated with reduced risks and improved outcomes in individuals with these disorders.^11–14^ These beneficial dietary patterns are thought to enhance brain plasticity and protect against neurodegeneration by providing essential nutrients and reducing inflammation.^15^

Genetic contributions to anxiety disorders and MDD, as well as dietary habits, have been extensively explored through twin studies and genome-wide association studies (GWASs). Heritability estimates indicate that genetics contribute approximately 30–60% to anxiety disorders^16–18^ and approximately 40% to MDD,^19^ indicating the crucial role of genetic components and their interactions with environmental factors in the pathogenesis of these disorders. Similarly, food preferences, eating behaviors, and susceptibility to certain dietary patterns, such as high-fat or sugar intake, have a heritability of approximately 20%-40%.^20^ Recent large-scale GWASs have identified 5 and 44 genetic loci associated with anxiety disorders and MDD, respectively,^21^^,^^22^ and have also revealed shared genetic predispositions between these disorders,^6^^,^^7^ highlighting their polygenic and overlapping characteristics. Similarly, large-scale GWASs on dietary intake and preferences have identified numerous loci,^23^^,^^24^ demonstrating that dietary preferences are influenced not only by environmental factors but also by genetic predispositions. These genetic findings enhance our understanding of the biological underpinnings of both mental disorders and dietary behaviors. Certain genetic variants associated with these mental disorders may also influence dietary choices, potentially explaining the genetic comorbidity and specificity of mental disorders. The development of polygenic risk scores (PRSs) has become a key tool^25^ in elucidating how multiple genetic factors for psychiatric disorders can cumulatively influence a wide range of human behaviors, including dietary preferences.

Given the observed associations between specific dietary patterns and these mental disorders, along with genetic similarities and distinctions between them, we hypothesized that PRSs for anxiety disorders and MDD would influence both common and specific dietary intakes, such as reduced intake of fruits and vegetables for shared influences. In this study, we focused on a cohort of 730 older adult outpatients who were diagnosed with or suspected of having common lifestyle-related diseases, such as hypertension, diabetes mellitus, and cancer. Patients with these physical conditions have a greater risk of anxiety disorders or MDD than do those without such diseases or the general population.^26–28^ The use of these participants allows for a comprehensive evaluation of how genetic predispositions for anxiety disorders and MDD impact dietary behaviors when influenced by physical health issues. We investigated the effects of PRSs for anxiety disorders and MDD on current dietary habits concerning 8 specific items in older patients with or suspected of having common lifestyle-related diseases. Additionally, we explored genetic correlations between the risk of anxiety disorders or MDD and specific dietary intake patterns in an independent general cohort from the UK Biobank (UKBB) to compare differences between the participating cohorts.

## Methods

### Targeting older adult patients

We recruited a total of 730 older adult outpatients aged 60 years and above who visited the Department of Clinical Laboratories, Iwata City Hospital, Shizuoka, Japan, for blood sampling during the recruitment period from 2003 to 2008^29–32^ (Table 1). These elderly patients were primarily diagnosed with or suspected of having lifestyle-related diseases, such as hypertension, diabetes mellitus, and cancer. All participants were unrelated, genetically homogeneous Japanese individuals, predominantly residing in the central region of Japan, namely, Tokai. The eligibility criteria for the patients included being over 60 years of age, ambulatory, and capable of verbal communication. Current or past contact with psychiatric services or the use of psychiatric medication was not assessed at the time of recruitment. Written informed consent was obtained from all patients. This study adhered to the ethical principles of the World Medical Association’s Declaration of Helsinki and was approved by the Institutional Review Boards of Iwata City Hospital and Hamamatsu University School of Medicine (21-8), Gifu University (2019-233), and Tokyo Institute of Psychiatry (now known as Tokyo Metropolitan Institute of Medical Science) (20-23(1)).

**Table 1:** Demographic information of 730 older adult patients diagnosed with or suspected of having any lifestyle-related diseases.

	**Cohort 1**	**Cohort 2**	**Total patients**
**Variable**	**(*n* = 300)**	**(*n* = 430)**	**(*n* = 730)**
Age (years)	73.3 ± 5.9	72.1 ± 6.8	72.6 ± 6.5
Sex (%male)	60.7	80.7	72.5
Body mass index	22.7 ± 3.4	22.5 ± 3.0	22.6 ± 3.2
Current alcohol drinker (%)	35.0	46.0	41.5
Current smoker (%)	16.7	21.2	19.3
Current hypertension (%)	44.7	34.4	38.6
Current diabetes mellitus (%)	24.7	12.8	17.5
Current cancer (%)	9.3	10.0	9.7
Past hypertension (%)	3.0	7.4	5.6
Past diabetes mellitus (%)	0.3	1.6	1.1
Past cancer (%)	10.7	15.6	13.6
**Current dietary habits**			
Miso soup (0-5 scale)	4.3 ± 1.3	4.3 ± 1.2	4.3 ± 1.3
Japanese tea (0-5 scale)	4.9 ± 0.8	4.8 ± 0.9	4.8 ± 0.8
Green and yellow vegetables (0-5 scale)	4.8 ± 0.6	4.3 ± 1.1	4.5 ± 0.9
Light-colored vegetables (0-5 scale)	4.8 ± 0.5	4.4 ± 1.0	4.6 ± 0.9
Fruits (0-5 scale)	4.0 ± 1.3	3.7 ± 1.5	3.8 ± 1.5
Pickles (0-5 scale)	3.3 ± 2.0	3.5 ± 1.9	3.4 ± 1.9
Meats (0-5 scale)	2.5 ± 1.1	2.3 ± 1.1	2.4 ± 1.1
Soybeans (0-5 scale)	4.4 ± 1.1	4.2 ± 1.2	4.3 ± 1.1

Cohort 1 comprised participants genotyped using the HumanCytoSNP v2.0 array (*n* = 300), and Cohort 2 comprised participants genotyped using the HumanCoreExome v1.0 array (*n* = 430). Complete demographic information was not obtained for all participants (height, *n* = 729; weight, *n* = 727; body mass index, *n* = 726; alcohol drinker, *n* = 729; light-colored vegetables, *n* = 729; fruits, *n* = 727; pickles, *n* = 728; meats, *n* = 729). The means ± SDs are shown.

### Current specific dietary intakes

The evaluation of 8 specific dietary intakes was conducted via a questionnaire.^29^^,^^30^^,^^32^ The questionnaire assessed the intake frequencies of the following dietary items: (1) miso soup, (2) Japanese tea, (3) green and yellow vegetables, (4) light-colored vegetables, (5) fruits, (6) pickles, (7) meats, and (8) soybeans. Responses were recorded on a 6-point scale: 0 (rarely); 1 (1-3 days a month); 2 (1-2 days a week); 3 (3-4 days a week); 4 (5-6 days a week); and 5 (daily). The correlations among these current dietary intakes were generally weak (*r* < 0.35), with the exception of a moderate correlation between the intakes of green and yellow vegetables and light-colored vegetables (*r* = 0.63).^32^

### Genotyping and quality control

A detailed overview of the genotyping and quality control (QC) methods used in this study has been provided previously.^31^ Briefly, DNA was extracted from peripheral venous blood samples. Patients were genotyped using 2 types of whole-genome genotyping arrays: HumanCytoSNP v2.0 (*n* = 300, Cohort 1) and HumanCoreExome v1.0 (*n* = 430, Cohort 2) BeadChips (Illumina, San Diego, CA, United States) (Table 1). During the QC process, samples with a genotype call rate less than 0.95 and single- nucleotide polymorphisms (SNPs) with a genotype call frequency less than 0.95 or a “cluster sep” (an index of genotype cluster separation) less than 0.1 were excluded.^31^ After this QC step, a total of 225 602 SNPs for HumanCytoSNP and 256 997 SNPs for HumanCoreExome were retained.^31^^,^^32^

### PRS calculations

To identify risk SNPs, along with their *P* values and effect sizes (odds ratios (ORs)) related to anxiety disorders and MDD, we utilized publicly available large-scale GWAS datasets for anxiety disorders and MDD as discovery samples.^21^^,^^22^ Based on these GWAS results of anxiety disorders^21^ and MDD,^22^ we calculated PRSs for our target patients. SNPs in linkage disequilibrium (LD) within our target patients were pruned via PLINK v1.9, applying a pairwise *r^2^* threshold of 0.25 and a window size of 200 SNPs. After the pruning process and the exclusion of SNPs located on sex and mitochondrial chromosomes, 72 223 independent SNPs for HumanCytoSNP and 68 313 independent SNPs for HumanCoreExome were retained. We calculated PRSs associated with these disorders at varying levels of significance within the discovery GWAS datasets using the following *P* value thresholds (*P_T cutoffs_*): *P_T_* < .01, *P_T_* < .05, *P_T_* < .1, *P_T_* < .2, *P_T_* < .5, and *P_T_* ≤ 1. For each target patient, the PRS was computed by summing the number of risk alleles (0, 1, or 2) multiplied by the effect size (logarithm of the OR) across all SNPs in the *P_T_*-SNP sets.

### Definitions of anxiety disorders and MDD in each discovery GWAS

#### Anxiety disorders

Anxiety disorders in the UKBB participants were identified through self-reported clinician-provided or probable lifetime diagnoses.^21^ Self-reporting clinician-provided anxiety disorders were based on individuals’ self-reports of receiving a lifetime professional diagnosis of 1 of the 5 core anxiety disorders: generalized anxiety disorder (GAD), social anxiety disorder, agoraphobia, specific phobia, or panic disorder. Probable lifetime anxiety disorder was defined according to a lifetime diagnosis of GAD based on the Diagnostic and Statistical Manual of Mental Disorders (DSM)-IV criteria, using anxiety-related questions from the Composite International Diagnostic Interview Short-form (CIDI-SF). Some participants met both criteria for inclusion for anxiety disorders.

#### Major depressive disorder

Major depressive disorder has been diagnosed in various cohorts, including the Psychiatric Genomics Consortium (PGC), UKBB, and iPSYCH, via the use of structured diagnostic instruments during assessments by trained interviewers, clinician-administered checklists, medical record reviews, or national registers. The diagnosis adhered to international consensus criteria, including the DSM-IV, ICD-9, or ICD-10, for a lifetime diagnosis of MDD or a variety of unique MDD assessment methods.^22^

### Statistical analyses

Statistical analyses were performed using IBM SPSS Statistics 28.0 software (IBM Japan, Tokyo, Japan). To evaluate the effects of PRSs for anxiety disorders and MDD at each *P_T_* on current dietary intake in our patients, linear regression analyses were performed with current dietary intake as the dependent variable, PRSs for these disorders as the independent variables, and age, sex, and array type as covariates. The adjusted *R^2^* value indicates the proportion of variance in current dietary intake explained by the PRSs. To isolate the variance specifically attributable to the PRSs, we subtracted the adjusted *R^2^* value for the covariates alone (age, sex, and array type) from that of the full models. Genetic SNP correlations (*r_g_*) among GWASs of anxiety disorders, MDD, and specific dietary habits were estimated using linkage disequilibrium score regression (LDSC) analysis.^6^^,^^33–38^ The nominal significance level was set at *p*<.05. Given that PRSs at each *P_T_* were highly correlated and not independent, *p* values derived from various *P_T_* values were not corrected for multiple comparisons. However, to avoid type I errors, a Bonferroni correction was applied, setting a *P* value threshold of *P* < 6.25 × 10^-3^ (*α* = 0.05/8 current dietary intake).

## Results

### Effects of PRSs for anxiety disorders and MDD on the frequency of 8 current dietary intakes

**Figure 1:**
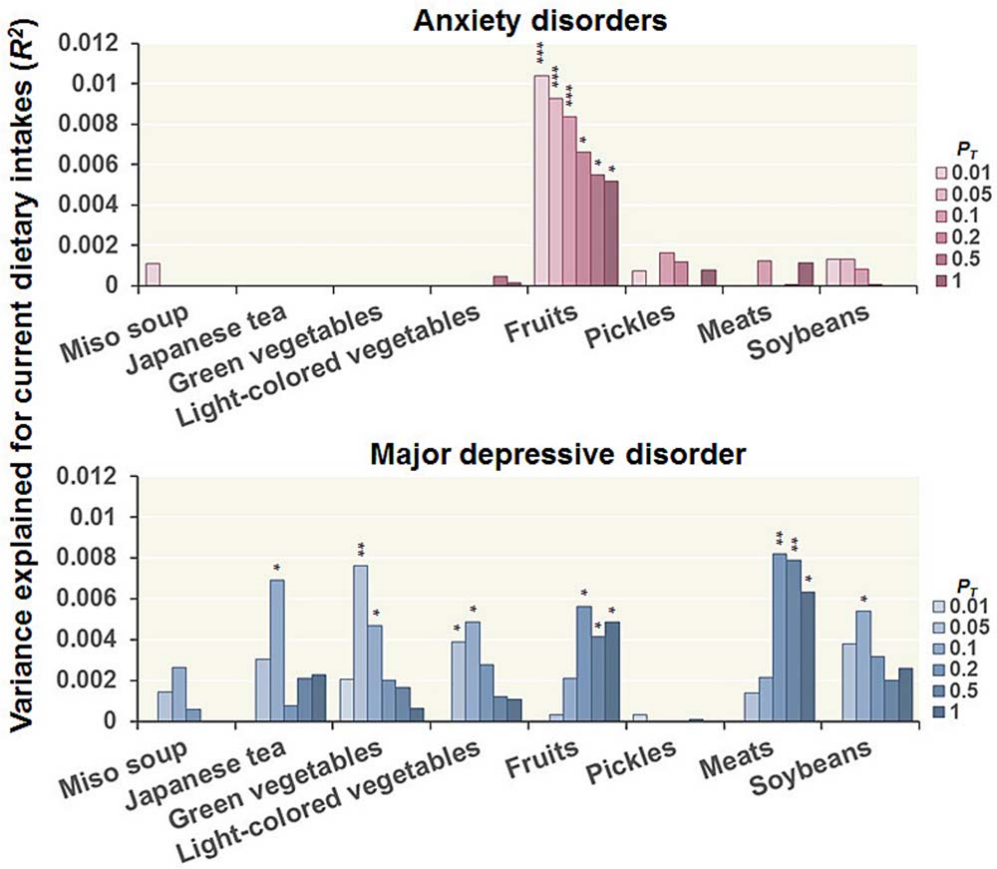
Effects of polygenic risk scores for anxiety disorders and major depressive disorder at various *P_T_* levels on 8 current dietary intakes. ^*^P < .05, ^**^P < .01, ^***^P < 6.25 × 10^-3^.

**Figure 2:**
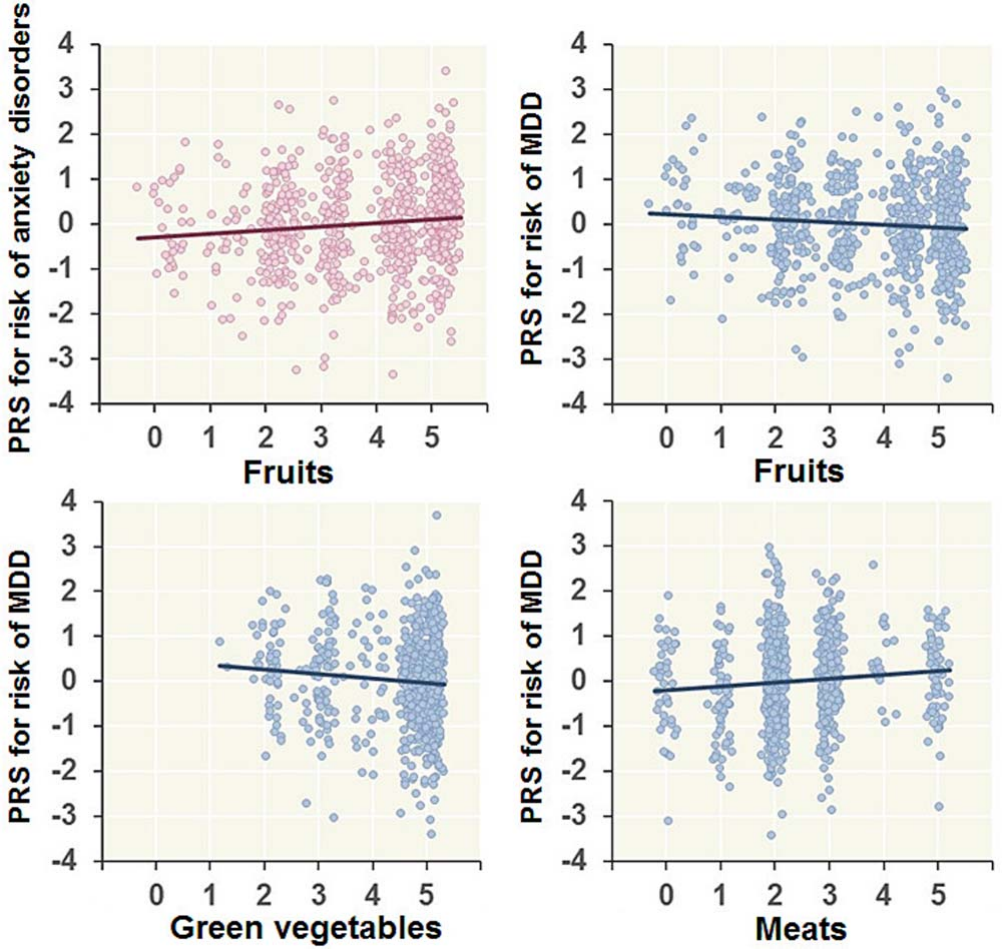
Correlations between polygenic risk scores for anxiety disorders and major depressive disorder and the current intake frequencies of fruits, green vegetables, and meats. The PRSs were *z*-standardized after adjusting for array type differences. Current dietary habits were adjusted for age and sex.

We investigated whether PRSs for anxiety disorders and MDD were correlated with the frequencies of current dietary intake of 8 specific items at varying *P_T_* levels (Figure 1). Among the 8 dietary items, the frequency of fruit intake was affected by PRSs for both anxiety disorders and MDD (*P* < .05), although the directions of the correlations were inconsistent across disorders. Higher PRSs for anxiety disorders were significantly correlated with increased fruit intake (maximum at *P_T_* < .01: *R^2^* = 0.010, *P* = 2.62 × 10^-3^), whereas higher PRSs for MDD were nominally correlated with decreased fruit intake (maximum at *P_T_* < .2: *R^2^* = 0.0056, *P* = .021) (Figure 2). No significant correlations were detected between the PRSs for anxiety disorders and the other 7 specific dietary intakes (*P* > .05). In contrast, PRSs for MDD were marginally negatively correlated with the intake frequencies of Japanese tea (maximum at *P_T_* < .1: *R^2^* = 0.0069, *P* = .014), green vegetables (Figure 2, maximum at *P_T_* < .05: *R^2^* = 0.0076, *P* = 7.82 × 10^-3^), light-colored vegetables (maximum at *P_T_* < .1: *R^2^* = 0.0049, *P* = .029), and soybeans (maximum at *P_T_* < .1: *R^2^* = 0.0054, *P* = .025) and were marginally positively correlated with meat intake frequency (Figure 2, maximum at *P_T_* < .2: *R^2^* = 0.0082, *P* = 8.02 × 10^-3^). There were no significant correlations between the PRSs for MDD and the intake frequencies of miso soup or pickles (*P* > .05). When participants were stratified by PRS deciles, mean fruit intake increased from 3.67 in the lowest decile to 4.08 in the highest decile for anxiety PRSs, whereas it decreased from 3.99 to 3.66 across corresponding deciles for MDD PRSs; Cohen’s *d* values relative to the lowest decile ranged from -0.29 to 0.32 for anxiety PRSs and from -0.31 to 0.01 for MDD PRSs (Supplementary Table 1).

Assuming a higher prevalence of anxiety disorders or MDD among patients diagnosed with or suspected of having any lifestyle-related diseases than in the general population,^26–28^ we adjusted for each type of current diagnostic status (+/-) of lifestyle-related diseases, such as hypertension, diabetes mellitus, or cancer, in the analyses. However, our findings were unaffected by each type of current diagnostic status and remained unchanged.

### Genetic correlations of anxiety disorders and MDD with fruit intake

**Figure 3:**
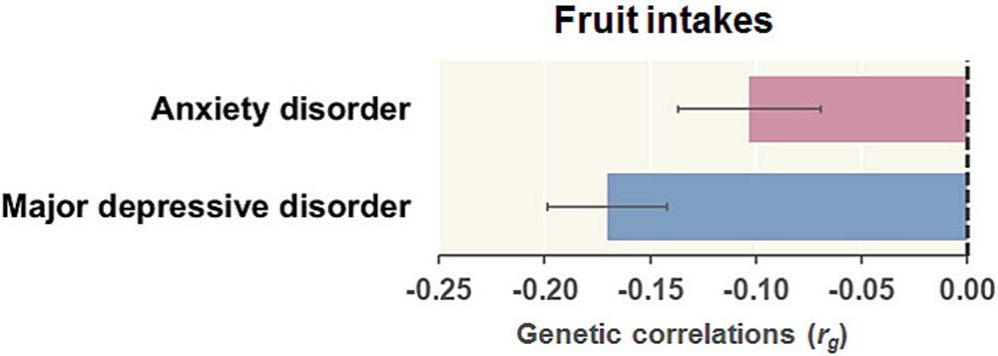
Genetic correlations (*r_g_*) of anxiety disorders and major depressive disorder with fruit intake. A negative *r_g_* indicates that a higher genetic risk of anxiety disorders or major depressive disorder is associated with lower fruit intake. The error bars represent the standard error.

Based on the opposing influences of PRSs for anxiety disorders and MDD on fruit intake in our older patients diagnosed with or suspected of having any lifestyle-related diseases, we further explored genetic correlations between GWASs of anxiety disorders and MDD and GWASs of fruit intake in UKBB general participants independent of our patients (https://www.ebi.ac.uk/gwas/)^23^ using LDSC analyses (Figure 3). Both anxiety disorders and MDD were genetically negatively correlated with fruit intake (Figure 3; anxiety disorders: *r_g_* = -0.10, *P* = 2.10 × 10^-3^; MDD: *r_g_* = -0.17, *P* = 2.01 × 10^-9^).

## Discussion

This study is the first to investigate the relationships between PRSs for anxiety disorders and MDD and current specific dietary intakes in older patients over 60 years of age who were diagnosed with, or suspected of having, lifestyle-related diseases. Higher PRSs for anxiety disorders were significantly correlated with increased fruit intake in these older patients. In contrast, higher PRSs for MDD were nominally correlated with decreased intake of fruits, Japanese tea, green vegetables, light-colored vegetables, and soybeans, and with increased intake of meats. Given that fruit intake frequency was inversely affected by PRSs for anxiety disorders and MDD, we explored genetic correlations between these disorders and fruit consumption in the independent UKBB general cohort. In this larger general cohort, a higher risk of these disorders was commonly genetically correlated with decreased fruit consumption. Importantly, the PRSs for anxiety disorders and MDD showed modest positive correlations in our patients (*r* ≈ 0.10-0.20), indicating shared genetic underpinnings that could influence dietary behaviors in a similar manner. These findings suggest that genetic factors for anxiety disorders might be related to compensatory healthy behaviors, such as fruit consumption, in response to the worry and anxiety related to suspected or diagnosed lifestyle-related diseases. This interpretation remains speculative because we did not directly assess anxiety symptoms, health-related worry, or behavioral change in this cohort. Alternative explanations, including cohort characteristics, age-related factors, and unmeasured socioeconomic or clinical variables, cannot be excluded.

Genetic predispositions for anxiety disorders and MDD could affect dietary habits throughout life. Additionally, environmental and psychological factors can influence dietary habits. Chronic physical issues might serve as environmental and psychological factors affecting these dietary habits. For individuals with a genetic predisposition for MDD, dietary habits may not change due to the loss of appetite associated with MDD, even if they have lifestyle-related diseases.^39^ In contrast, individuals with a genetic predisposition for anxiety disorders may experience anxiety and worry about physical issues, which could serve as environmental and psychological factors that prompt these individuals to review and change their dietary habits.^26^ Therefore, genetic factors associated with anxiety disorders might be related to compensatory dietary behaviors in response to anxiety and worry related to physical issues.

Although direct evidence linking anxiety disorders to healthier dietary choices is limited, prior research in health psychology suggests that heightened perceptions of health threat or health anxiety can motivate engagement in preventive health behaviors. For example, individuals with greater perceived susceptibility to illness have been reported to engage more frequently in health-promoting behaviors, consistent with theoretical frameworks such as the Health Belief Model.^40^^,^^41^ In adults with chronic physical illness, psychological factors such as perceived meaning and controllability of life and emotional well-being have been associated with engagement in health-promoting behaviors, including dietary and other lifestyle modifications.^42^ These findings offer additional support for interpreting the present results, while emphasizing that direct causal pathways remain to be established.

We found that higher PRSs for MDD were marginally correlated with decreased intake of Japanese tea, green and light-colored vegetables, fruits, and soybeans, and with increased intake of meats in patients diagnosed with, or suspected of having, lifestyle-related diseases. However, the PRSs for anxiety disorders were not significantly correlated with such unhealthy dietary patterns in these patients. Considering the high genetic correlations between MDD and anxiety disorders^6^^,^^7^ and the negative genetic correlations between these disorders and fruit consumption, as shown in Figure 3, the PRSs for anxiety disorders may be related to unhealthy dietary patterns in patients without lifestyle-related diseases or in the general population.

Individuals with a genetic predisposition for anxiety disorders or MDD might exhibit unhealthy dietary behaviors for several reasons. First, anxiety disorders and MDD can lead to emotional eating or a loss of appetite, both of which disrupt normal dietary patterns. Emotional eating, where individuals consume food in response to stress or negative emotions, is a common coping mechanism that often involves high-calorie, low-nutrient foods.^43^ Additionally, the psychological impact of these disorders can reduce motivation and energy levels, making meal preparation and adherence to healthy diets less appealing.^44^ Genetic predispositions can influence neurotransmitter systems involved in both mood regulation and appetite control, such as the serotonin pathway, which is known to be involved in both depression and appetite regulation.^45^ Furthermore, chronic stress associated with anxiety can activate the hypothalamic–pituitary–adrenal (HPA) axis, affecting hunger and satiety signals and thereby contributing to unhealthy dietary choices.^46^ Therefore, the interactions among genetic, psychological, and neurobiological factors can affect dietary behaviors in individuals with anxiety disorders or MDD.

There are several limitations to the interpretation of our findings. Importantly, the proportions of variance explained by the PRSs were small (maximum *R^2^* ≈ 1%), indicating that these associations represent subtle population-level tendencies rather than effects with strong predictive value for individual dietary behaviors. Our cohort, consisting of older patients diagnosed with or suspected of having any lifestyle-related diseases, may not represent the general population or patients specifically with anxiety disorders or MDD. This limitation potentially restricts the generalizability of our findings. Not all types of dietary items were comprehensively covered by our questionnaire, which included only 8 dietary items and therefore may not fully capture overall dietary patterns, omitting others such as fish, rice, grains, eggs, milk, snacks, and nuts. Although we examined the effects of genetic predispositions for anxiety disorders and MDD on dietary behaviors in older patients, confounding factors, such as IQ, income, lifestyle, and unmeasured environmental influences, may also contribute to these associations.

In conclusion, we investigated whether genetic predispositions for anxiety disorders and MDD were associated with current dietary intake patterns in older patients with lifestyle-related diseases. Despite a similar genetic background between anxiety disorders and MDD, the PRSs for anxiety disorders and MDD were associated with distinct current dietary intake patterns, including increased and decreased fruit consumption, respectively. Conversely, both disorders are commonly genetically correlated with decreased fruit intake in the independent general population. Our findings suggest that genetic factors related to anxiety disorders might play a role in compensatory healthy dietary behaviors against anxiety and worry related to physical health issues. These associations were modest in magnitude and should be interpreted as subtle population-level effects rather than clinically meaningful predictors of individual dietary behaviors.

## Supplementary Material

Supplementary_Table_1_pyag014

## Data Availability

The data are not publicly available because they contain information that could compromise research participant privacy/consent.
