# Supplementary material for: Genetic predispositions for anxiety disorders and major depressive disorder affect current dietary habits in older patients with lifestyle-related diseases
Source: Int J Neuropsychopharmacol. 2026 Mar 21;29(4):pyag014. doi: 10.1093/ijnp/pyag014 (PMC13076931; doi:10.1093/ijnp/pyag014)
Supplement: Supplementary_Table_1_pyag014 [file supplementary_table_1_pyag014.docx]

**Supplementary Table 1.** Mean (SD) fruit intake by deciles of polygenic risk scores (PRSs) for anxiety disorders and major depressive disorder (MDD), with Cohen’s *d* values relative to the lowest decile.

| PRS for anxiety disorders (decile) | *n* | Mean | SD | Cohen’s *d* (vs lowest decile) |
| --- | --- | --- | --- | --- |
| 1 (lowest) | 73 | 3.67 | 1.32 | - (Reference) |
| 2 | 73 | 3.26 | 1.50 | -0.29 |
| 3 | 73 | 3.70 | 1.47 | 0.02 |
| 4 | 72 | 3.66 | 1.47 | -0.01 |
| 5 | 73 | 4.07 | 1.27 | 0.30 |
| 6 | 73 | 3.87 | 1.42 | 0.14 |
| 7 | 73 | 3.94 | 1.44 | 0.19 |
| 8 | 72 | 3.72 | 1.46 | 0.03 |
| 9 | 73 | 4.00 | 1.48 | 0.23 |
| 10 (highest) | 72 | 4.08 | 1.20 | 0.32 |

| PRS for MDD (decile) | *n* | Mean | SD | Cohen’s *d* (vs lowest decile) |
| --- | --- | --- | --- | --- |
| 1 (lowest) | 72 | 3.99 | 1.30 | - (Reference) |
| 2 | 73 | 4.00 | 1.35 | 0.01 |
| 3 | 73 | 3.83 | 1.36 | -0.12 |
| 4 | 73 | 3.86 | 1.33 | -0.10 |
| 5 | 73 | 4.00 | 1.31 | 0.01 |
| 6 | 73 | 3.58 | 1.46 | -0.30 |
| 7 | 71 | 3.77 | 1.56 | -0.15 |
| 8 | 73 | 3.57 | 1.43 | -0.31 |
| 9 | 73 | 3.72 | 1.52 | -0.19 |
| 10 (highest) | 73 | 3.66 | 1.51 | -0.23 |

PRSs were *z*-standardized after adjusting for array type differences. Fruit intake was adjusted for age and sex. Cohen’s *d* values represent standardized mean differences between each decile and the lowest decile. Higher positive values indicate greater fruit intake relative to the lowest PRS group.
